# Increased Levels of Circulating Angiogenic Cells and Signaling Proteins in Older Adults With Cerebral Small Vessel Disease

**DOI:** 10.3389/fnagi.2021.711784

**Published:** 2021-09-28

**Authors:** Arunima Kapoor, Aimée Gaubert, Anisa Marshall, Irene B. Meier, Belinda Yew, Jean K. Ho, Anna E. Blanken, Shubir Dutt, Isabel J. Sible, Yanrong Li, Jung Yun Jang, Adam M. Brickman, Kathleen Rodgers, Daniel A. Nation

**Affiliations:** ^1^Department of Psychological Science, University of California, Irvine, Irvine, CA, United States; ^2^Institute for Memory Impairments and Neurological Disorders, University of California, Irvine, Irvine, CA, United States; ^3^Department of Psychology, University of Southern California, Los Angeles, CA, United States; ^4^Department of Neurology, Taub Institute for Research on Alzheimer’s Disease and the Aging Brain, Columbia University, New York, NY, United States; ^5^Chione GmbH, Binz, Switzerland; ^6^Center for Innovation in Brain Science, Department of Pharmacology, The University of Arizona, Tucson, AZ, United States

**Keywords:** vascular endothelial growth factor, endothelial progenitor cells, cerebral microvascular pathology, cerebral small vessel disease, aging, vascular dementia, dementia

## Abstract

**Background:** Cerebral small vessel disease (SVD) is associated with increased risk of stroke and dementia. Progressive damage to the cerebral microvasculature may also trigger angiogenic processes to promote vessel repair. Elevated levels of circulating endothelial progenitor cells (EPCs) and pro-angiogenic signaling proteins are observed in response to vascular injury. We aimed to examine circulating levels of EPCs and proangiogenic proteins in older adults with evidence of SVD.

**Methods:** Older adults (ages 55–90) free of dementia or stroke underwent venipuncture and brain magnetic resonance imaging (MRI). Flow cytometry quantified circulating EPCs as the number of cells in the lymphocyte gate positively expressing EPC surface markers (CD34+CD133+CD309+). Plasma was assayed for proangiogenic factors (VEGF-A, VEGF-C, VEGF-D, Tie-2, and Flt-1). Total SVD burden score was determined based on MRI markers, including white matter hyperintensities, cerebral microbleeds and lacunes.

**Results:** Sixty-four older adults were included. Linear regression revealed that older adults with higher circulating EPC levels exhibited greater total SVD burden [β = 1.0 × 10^5^, 95% CI (0.2, 1.9), *p* = 0.019], after accounting for age and sex. Similarly, a positive relationship between circulating VEGF-D and total SVD score was observed, controlling for age and sex [β = 0.001, 95% CI (0.000, 0.001), *p* = 0.048].

**Conclusion:** These findings suggest that elevated levels of circulating EPCs and VEGF-D correspond with greater cerebral SVD burden in older adults. Additional studies are warranted to determine whether activation of systemic angiogenic growth factors and EPCs represents an early attempt to rescue the vascular endothelium and repair damage in SVD.

## Introduction

Cerebral small vessel disease (SVD) occurs commonly with advancing age and is associated with increased risk of cognitive impairment, vascular dementia and Alzheimer’s disease, contributing to up to 45% of all cases of dementia (Gorelick et al., 2011; Lee et al., 2016). Small vessel changes often remain asymptomatic, but are evident on brain magnetic resonance imaging (MRI) (Wardlaw et al., 2019) and believed to result from pathological changes in the perforating cerebral arteries, penetrating arterioles, capillaries, and venules (Pantoni, 2010). Microvascular function is crucial to addressing cerebral metabolic demands, clearing waste products and maintaining the blood-brain barrier (Zlokovic, 2008; Chabriat et al., 2009; Wardlaw et al., 2013a, 2019; Pettersen et al., 2017; Nation et al., 2019). Reduced blood flow and hypoxia due to degradation of small blood vessels may trigger angiogenesis—the process of blood vessel growth—as a compensatory mechanism (Quick et al., 2021). While it is still debated whether the process of angiogenesis is beneficial or detrimental in the context of vascular brain injury, animal models and studies of large vessel disease show increased angiogenesis in response to vascular injury (Adamczak and Hoehn, 2015).

One feature of angiogenesis is the mobilization of cells that can repair the vasculature. Endothelial progenitor cells (EPCs) represent a heterogeneous cell population known to be mobilized in response to vascular-endothelial damage to promote vessel repair and regenesis (Medina et al., 2017). Increased levels of circulating EPCs at day 7 post-intracerebral hemorrhage predict improved functional outcome at 12 months (Pías-Peleteiro et al., 2016) and mobilization of EPCs has been observed in response to cerebral large vessel disease (Sobrino et al., 2007). Animal mouse models of ischemic stroke suggest that transplantation of *in vitro* cultured EPCs attenuates blood–brain barrier leakage, tight junction protein degradation, and neurological deficits (Geng et al., 2017; Kong et al., 2018).

Similarly, damage to the cerebral vasculature can trigger the release of signaling proteins that are involved in angiogenesis. The vascular endothelial growth factor (VEGF) family proteins, as well as other angiogenic factors, are also known to induce endothelial and neuronal remodeling after stroke (Hayakawa et al., 2017). Levels of these proteins are elevated after ischemic insult in brain tissue and serum in rodent models and in humans (Issa et al., 1999; Zhang et al., 2000; Prodjohardjono et al., 2020). However, few previous studies have evaluated whether changes in circulating angiogenic cells and proteins also occur in response to progressive and insidious cerebral SVD changes which commonly occur with advancing age.

The current study evaluated whether circulating levels of EPCs and pro-angiogenic proteins are associated with SVD burden in older adults who were otherwise neurologically healthy, with no history of clinical stroke or dementia. Consistent with prior studies, we hypothesized that higher levels of circulating angiogenic proteins and EPCs would be associated with greater cerebral SVD burden.

## Methods

### Participants

Participants were recruited from the community and included if they were 55 years of age or older, independently living with no history of clinical stroke, dementia or other systemic or neurological illness that may impact central nervous system function. History of vascular risk factors, including hypertension, body mass index, dyslipidemia, diabetes, as well as history of other medical illnesses, was determined by clinical interview. This study was approved by the local Institutional Review Board; all participants gave informed consent and underwent blood draw and brain MRI.

### Endothelial Progenitor Cell Quantification

Venipuncture was performed after an overnight fast. EPCs were quantified with flow cytometry. Peripheral blood mononuclear cells (PBMCs) were isolated by density gradient centrifugation and washed twice with DPBS + 2% FBS at 120 × *g* for 10 min and 300 × *g* for 8 min at room temperature. Fluorescently labeled antibodies to EPC surface antigens were utilized (Nation et al., 2018). 1 million cells were transferred to an unstained tube blank, and the remainder was transferred to the stained tube, where 5 μL of Human BD Fc Block (BD Biosciences) was added and incubated for 10– 15 min. An additional 1 μL of each of the following antibodies were then added: (Gorelick et al., 2011) CD34-PE-Vio770 (clone: AC136, Miltenyi Biotec), (Lee et al., 2016) CD133-VioBright FITC (clone: AC133, Miltenyi Biotec), (Wardlaw et al., 2019) PerCP/Cy5.5-CD309 (clone: 7D4-6, BioLegend). Tubes were incubated in the dark for 30 min at 4°C. Samples were washed with 3 mL PBS, centrifuged at 300 × *g* for 8 min, and fixed with 2% formaldehyde in PBS until analysis. Compensation controls were conducted using AbC Total Antibody Compensation Bead Kit (Thermo Fisher Scientific). Samples were acquired on a BD LSR II flow cytometer and analyzed on FlowJo software. EPCs were defined as CD34+CD133+CD309+ cells (Medina et al., 2017). Circulating EPCs were quantified by flow cytometry as the number of cells in the lymphocyte gate positively expressing EPC surface markers (CD34+CD133+CD309+). CD34+CD133+CD309+ cell concentrations were calculated as a percentage of lymphocytes.

### APOE Genotyping

Apolipoprotein E (APOE) genotyping was conducted on the blood cell pellet fraction obtained from plasma separation. DNA was isolated from the pellet fraction using the PureLink Genomic DNA Mini Kit (Thermo Fisher Scientific). Genotyping was conducted on isolated DNA using the TaqMan SNP Genotyping Assay (Thermo Fisher Scientific) on an Applied Biosystems 7300 Real Time PCR System. APOE gene SNPs were assessed for dbSNP IDs rs429358 and rs7412. Allelic discrimination was conducted using the included qPCR software. The APOE-ε4 allele was designated as rs429358-C + rs7412-C.

### Angiogenic Protein Levels

Levels of angiogenic proteins in plasma were determined using the Meso Scale Discovery V- PLEX Human Biomarker 40-Plex Kit Angiogenesis Panel 1 (VEGF-A, VEGF-C, VEGF-D, Tie-2, Flt-1, PIGF, and bFGF), following manufacturer’s protocol without modification. Briefly, we utilized the MSD Multi-Spot 96-well 7-spot plate pre-coated with capture antibodies. Plates were washed three times with at least 150 μl/well of wash buffer. 50 μl of diluted plasma samples per well were added for the angiogenesis panel and incubated at room temperature with shaking for 2 h. Plates were then washed 3 times with at least 150 μl/well of washing buffer. 25 μl of detection antibody solution was added to each well and incubated at room temperature with shaking for 2 h. Plates were then washed three times again. 150 μl of 2X Read Buffer T was added to each well. Plates were then analyzed on an MSD instrument. Values below the lower limit of detection (LLOD) were replaced with zero.

### Cerebral Small Vessel Disease Quantification

All participants underwent a comprehensive neuroimaging protocol as previously described (Nation et al., 2018). The following sequences were examined for the current analysis: 3D T1-weighted anatomical scan for qualitative assessment of brain structures and abnormalities, T2-weighted scan for identification and differentiation of lacunes, fluid-attenuated inversion recovery (T2-FLAIR) for the evaluation of white matter hyperintensities, and T2^∗^-weighted imaging for assessment of cerebral microbleeds (Pantoni, 2010; Gorelick et al., 2011; Lee et al., 2016; Wardlaw et al., 2019). MRI markers were identified and scored in accordance with established neuroimaging standards for SVD (Wardlaw et al., 2013b). To determine total MRI SVD burden, all imaging markers were combined using a SVD score (amended version) recently developed by Amin Al Olama et al. (2020), which ranges from 0 to 7 and includes grading of white matter hyperintensities (0–3; using the Fazekas scale), number of lacunes (0–3; 0 = no lacunes, 1 = 1–2, 2 = 3–5, 3 = > 5) and presence of microbleeds (0–1; 0 = absent, 1 = present).

### Statistical Analyses

All analyses were performed using IBM SPSS Statistics 27 and R Version 3.6.1. Data were initially screened for extreme values (outliers) defined as values greater than 5 standard deviations from the mean and values that were unduly affecting regression parameter estimates based on measures of distance or influence. Two participants had extreme values for CD34+CD133+CD309+ cell counts, two participants had extreme values for VEGF-C and one participant had an extreme value for VEGF-D and were excluded from analyses. We examined the relationship between circulating EPCs as well as angiogenic proteins (independent predictors) and SVD score (dependent outcome), independently and after adjusting for age and sex. In addition, we examined any significant effects of APOE4 carrier status on the relationship between angiogenic circulating cells as well as proteins and SVD burden. Significance threshold was set at *p* < 0.05 for all analyses.

## Results

A total of 64 participants were included in the analysis. Age of study participants ranged from 55 to 90 years [Mean (*M*) = 69.8, standard deviation (SD) = 7.3], education ranged from 6 to 20 years [*M* = 15.8, SD = 2.8] and 40.6% were male (Table 1). Levels of circulating angiogenic proteins can be found in Table 2.

**TABLE 1:** Participant characteristics, demographics, and vascular risk factors.

	***N* = 64**
Age (years), *M* (SD)	69.8 (7.3)
Sex male, *n* (%)	26 (40.6)
Education (years), M (SD)	15.8 (2.8)
APOE4 Carrier, *n* (%)	20(33.3%)
Hypertension, *n* (%)	26 (40.6)
Dyslipidemia, *n* (%)	33 (51.6)
Diabetes, *n* (%)	6 (9.4)
Smoking history, *n* (%)	26 (40.6)
TIA, *n* (%)	1 (1.6)
Cardiovascular disease, *n* (%)	5 (7.9)
Atrial fibrillation, *n* (%)	3 (4.8)
Left ventricular hypertrophy, *n* (%)	1 (1.6)

*History of cardiovascular disease, atrial fibrillation, and left ventricular hypertrophy was collected for 63 (98.4%) participants and missing for one participant. APOE4 carrier status was missing for four participants and available for 60 participants.

**TABLE 2:** Angiogenic protein levels.

	***N***	**Mean**
VEGF (pg/mL), *M* (SD)	64	112.08 (116.97)
VEGF-C (pg/mL), *M* (SD)	64	91.53 (154.36)
VEGF-D (pg/mL), *M* (SD)	64	849.76 (818.72)
Tie-2 (pg/mL), *M* (SD)	64	3180.27 (1106.89)
Flt-1 (pg/mL), *M* (SD)	64	57.04 (21.28)
PlGF (pg/mL), *M* (SD)	35	3.08 (1.29)
bFGF (pg/mL), *M* (SD)	35	2.90 (2.70)

### Cerebral Small Vessel Disease Burden

**FIGURE 1:**
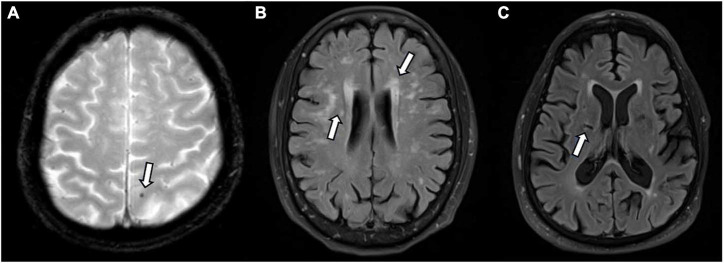
Small vessel disease as identified on magnetic resonance imaging (MRI) by white arrows. **(A)** Microbleed identified on T2*; **(B)** white matter hyperintensities identified on FLAIR; **(C)** lacune on FLAIR.

Small vessel disease markers were evident (SVD score > 1) in 29 (45.3%) participants (Figure 1). SVD scores ranged from 0 to 4 (*M* = 1.6, SD = 1.0). Microbleeds were identified in 5 (7.8%) participants, and small lacunes in 8 (12.5%) participants. White matter hyperintensities were identified in majority of participants; 36 (56.3%) displayed mild white matter hyperintensity burden (Fazekas 1), 18 (28.1%) displayed moderate burden (Fazekas 2) and 6 (9.4%) showed severe burden (Fazekas 3).

### Association Between Endothelial Progenitor Cells and Small Vessel Disease Burden

Endothelial progenitor cell count (CD34+CD133+CD309+ count per lymphocyte) and SVD score was available for 52 participants and 2 outliers were removed; 50 participants were therefore included in this analysis. Mean EPC count was 2.7 × 10^–6^ CD34+CD133+CD309+ cells/lymphocyte (SD = 3.4 × 10^–6^). Linear regression revealed that higher levels of circulating EPCs was associated with greater SVD burden, even after accounting for age and sex in multiple regression [β = 1.0 × 10^5^, 95% CI (0.2, 1.9), *p* = 0.019]. The relationship remained significant even after adjusting for APOE4 carrier status and a significant positive effect of APOE4 carrier status was observed, with APOE4 carriers having greater SVD burden [*N* = 48; β = 0.57, 95% CI (0.01, 1.13), *p* = 0.046; Table 3].

**TABLE 3:** Association between EPCs and small vessel disease.

**Variable**	**Unstandardized**	**Coefficients**	**Standardized**	**Sig.**	**95% confidence interval for B**	
	**B**	**Std. error**	**B**		**Lower bound**	**Upper bound**
Age (years)	0.004	0.019	0.033	0.838	−0.035	0.043
Sex (male)	−0.153	0.269	−0.080	0.572	−0.696	0.389
APOE4 (carrier)	0.568	0.276	0.291	0.046	0.011	1.125
Circulating EPCs (CD34+CD133+CD309+/lymphocyte)	105447.5	41519.0	0.394	0.015	21716.5	189178.4

*Dependent variable: SVD score.*

### Association Between Pro-angiogenic Proteins and Small Vessel Disease Burden

We specifically examined the relationship of VEGF-A, VEGF-C, VEGF-D, Tie-2, and Flt-1 with SVD score. Values were available for all 64 participants, however, two participants had extreme values for VEGF-C and one participant had an extreme value for VEGF-D and were excluded from the current analyses. Simple linear regression revealed a positive relationship only between circulating VEGF-D and total SVD score, which remained significant in multiple regression controlling for age and sex [β = 0.001, 95% CI (0.000, 0.001), *p* = 0.048]. The relationship remained significant even after adjusting for APOE4 carrier status (Table 4).

**TABLE 4:** Association between VEGF-D and small vessel disease.

**Variable**	**Unstandardized**	**Coefficients**	**Standardized**	**Sig.**	**95% confidence interval for B**	
	**B**	**Std. error**	**B**		**Lower bound**	**Upper bound**
Age (years)	0.031	0.018	0.236	0.092	−0.005	0.067
Sex (male)	−0.078	0.255	−0.039	0.761	−0.589	0.434
APOE4 (carrier)	0.330	0.283	0.159	0.249	−0.237	0.898
Circulating VEGF-D (pg/mL)	0.001	0.000	0.257	0.048	0.000	0.001

*Dependent variable: SVD score.*

## Discussion

Mobilization of EPCs and release of angiogenic growth factors has been observed in response to cerebral large vessel disease (Sobrino et al., 2007). Consistent with such studies, our findings indicate increased levels of circulating EPCs and angiogenic proteins in the presence of cerebral SVD. Specifically, circulating numbers of CD34+CD133+CD309+ cells and levels of VEGF-D protein correlated with greater cerebral SVD burden based on white matter hyperintensities, cerebral microbleeds and lacunes. SVD markers were evident on MRI in almost half our sample, yet participants did not have major cognitive dysfunction at the time of assessment, suggesting that the correlation between cerebral SVD and these angiogenic factors may be observed during the preclinical disease stage. While the current study was correlational, precluding causal inference, our findings are consistent with the hypothesis that EPCs and angiogenic signaling proteins may be increased in circulation in response to cerebral SVD (Nation et al., 2018).

Studies focused on clinically symptomatic patients with more severe white matter changes, cognitive impairment and dementia indicate a limited pool of EPCs may be exhausted with the emergence and progression of clinical symptomatology (Iadecola, 2013). Based on these data and the present study findings, we hypothesize that increased circulating EPC and VEGF-D levels in asymptomatic older adults may represent preclinical markers of early-stage cerebral small vessel injury, and that later exhaustion of the EPC pool may coincide with progressive cognitive decline (Lee et al., 2009). Elevation of VEGF in the presence of cognitive impairment is supported by prior research (Mahoney et al., 2021), although the literature suggests that EPC levels deplete in correspondence with the decline in cognitive function (Lee et al., 2009). If our hypothesized model is supported, the potential for circulating EPC and VEGF-D levels biomarkers for early-stage cerebral SVD changes warrants further research. Moreover, animal studies suggest that transplantation of EPCs may attenuate blood brain barrier breakdown (Geng et al., 2017), tight junction protein degradation, and neurological deficits (Geng et al., 2017; Kong et al., 2018), suggesting that EPCs may also offer potential therapeutic opportunities. Similarly, *in vitro* studies suggest that VEGF may support EPC differentiation and vascular repair (Li et al., 2017) but additional studies are needed.

Increased levels of VEGF-D have been associated with atrial fibrillation, ischemic stroke and heart failure (Borné et al., 2018; Berntsson et al., 2019) and elevated levels are known to predict mortality in patients with coronary artery disease (Wada et al., 2020). VEGF-D is a secreted glycoprotein and one of five members of the VEGF family with high angiogenic and lymphangiogenic potential (Lohela et al., 2009). Recent animal and *in vitro* studies also suggest a role for VEGF-D in dendrite maintenance required for memory formation and other cognitive processes (Mauceri et al., 2011, 2020; Stacker and Achen, 2018). It remains unclear whether circulating EPCs may play related roles in central nervous system functions. In the present study, we demonstrate an association between circulating VEGF-D and SVD in older adults, suggesting that angiogenic and lymphangiogenic processes may be taking places. Prior studies have demonstrated a role for VEGF-A in EPC recruitment to stimulate vasculogenesis, however, no prior studies have examined the relationship between VEGF-D and EPCs, making it challenging to understand the relationship and directionality between the two based on current literature. Further studies are warranted to confirm these findings and uncover potential mechanisms.

Whether the mobilization of EPCs and angiogenic factors to promote angiogenesis is beneficial or detrimental in SVD warrants further attention. Angiogenic processes, including endothelial barrier function and cell connections that may be weakened during repair and remodeling, may lead to inflammation and increased blood–brain barrier permeability (Adamczak and Hoehn, 2015). The involvement and impact of angiogenic cells and signaling proteins in repairing cerebral SVD—often a progressive form of vascular damage which worsens with age—may be important in understanding cognitive decline in older adults.

Limitations of this study include the limited sample size and cross-sectional design. Further studies may elucidate the predictive value of these angiogenic biomarkers. Moreover, additional angiogenic markers warrant further attention. We utilized the MSD angiogenesis biomarker panel in this study; however, numerous other proteins play a significant role in mediating angiogenesis and advanced techniques could be further utilized to identify and examine additional markers of angiogenesis. Future studies could also examine the relationship between inflammation and EPCs, given that recent studies suggest that inflammation may influence EPC function (Naserian et al., 2020; Nouri Barkestani et al., 2021).

Another limitation is the lack of specificity and varying definitions of EPCs in current literature. Gating for flow cytometry and calculation of EPCs also remains inconsistent. We utilized the consensus statement and recent studies to define EPCs as CD34+CD133+CD309+ cells (Medina et al., 2017). However, inclusion of cell markers such as CD45 remains inconsistent (Medina et al., 2017) and commonly employed methods to quantify EPCs assume a high risk of cell loss. Additional studies are needed to better understand and quantify the EPC population. The findings of this study provide further insight into the potential role of angiogenesis in microvascular pathology of the aging brain.

## Data Availability Statement

The raw data supporting the conclusions of this article will be made available by the authors, upon reasonable request.

## Ethics Statement

The studies involving human participants were reviewed and approved by the University of Southern California and University of California, Irvine. The patients/participants provided their written informed consent to participate in this study.

## Author Contributions

DN contributed to the study design. AK and DN contributed to analyzing the data and drafting the manuscript. AG, AM, IM, BY, JH, AEB, SD, IS, YL, JJ, AMB, and KR contributed to data collection. All authors contributed to manuscript revision, read, and approved the submitted version.

## Conflict of Interest

IM was employed by company Chione GmbH. The remaining authors declare that the research was conducted in the absence of any commercial or financial relationships that could be construed as a potential conflict of interest.

## Publisher’s Note

All claims expressed in this article are solely those of the authors and do not necessarily represent those of their affiliated organizations, or those of the publisher, the editors and the reviewers. Any product that may be evaluated in this article, or claim that may be made by its manufacturer, is not guaranteed or endorsed by the publisher.
